# Supplementary material for: Characteristics and Outcomes of Colorectal Cancer Patients Cared for by the Multidisciplinary Team in the Reggio Emilia Province, Italy
Source: Cancers (Basel). 2024 Jun 28;16(13):2390. doi: 10.3390/cancers16132390 (PMC11240821; doi:10.3390/cancers16132390)
Supplement: Supplementary file 1 [file cancers-16-02390-s001.zip › cancers-3038162-supplementary.pdf]

Table S1. Reggio Emilia Cancer Registry, years 2017-2018. Cox Regression analysis in the entire cohort, and by MDT

	Entire cohort		MDT Yes		MDT No	
	**HR**	**95% CI**	**HR**	**95% CI**	**HR**	**95% CI**
**Age**						
<70	1.0	Ref.	1.0	Ref.	1.0	Ref.
70+	3.0	2.2–4.0	3.0	1.9–4.7	3.1	2.0–4.6
**Year**						
2017	1.0	Ref.	1.0	Ref.	1.0	Ref.
2018	1.3	1.0–1.6	1.4	1.0–2.1	1.5	1.1–2.1
**Site**						
Colon	1.0	Ref.	1.0	Ref.	1.0	Ref.
Rectum	1.0	0.8–1.3	1.2	0.8–1.8	0.8	0.5–1.2
**Sex**						
Male	1.0	Ref.	1.0	Ref.	1.0	Ref.
Female	1.1	0.8–1.3	1.2	0.8–1.7	0.9	0.7–1.3
**Stage**						
I	1.0	Ref.	1.0	Ref.	1.0	Ref.
II	1.4	0.9–2.2	1.5	0.8–2.7	1.1	0.5–2.2
III	3.4	2.2–5.1	3.0	1.7–5.3	3.2	1.7–6.1
IV	11.0	7.2–16.7	9.3	5.2–16.8	10.5	5.4–20.3
